# Genetic Polymorphisms of *ENPP2* Are Possibly Associated with Pain Severity and Opioid Dose Requirements in Patients with Inflammatory Pain Conditions: Clinical Observation Study

**DOI:** 10.3390/ijms24086986

**Published:** 2023-04-10

**Authors:** Rikuhei Tsuchida, Daisuke Nishizawa, Ken-ichi Fukuda, Tatsuya Ichinohe, Kuniyuki Kano, Makoto Kurano, Kazutaka Ikeda, Masahiko Sumitani

**Affiliations:** 1 Department of Anesthesiology and Pain Relief Center, The University of Tokyo Hospital, Hongo 7-3-1, Bunkyoku, Tokyo 113-8655, Japan; 2 Addictive Substance Project, Tokyo Metropolitan Institute of Medical Science, Kami Kitazawa 2-1-6, Setagayaku, Tokyo 156-0057, Japan; 3 Department of Oral Health and Clinical Science, Tokyo Dental College, Kanda Misakichou 2-9-18, Chiyodaku, Tokyo 101-0061, Japan; 4 Department of Dental Anesthesiology, Tokyo Dental College, Kanda Misakichou 2-9-18, Chiyodaku, Tokyo 101-0061, Japan; 5 Department of Health Chemistry, Graduate School of Pharmaceutical Sciences, The University of Tokyo, Hongo 7-3-1, Bunkyoku, Tokyo 113-8655, Japan; 6 Department of Clinical Laboratory Medicine, The University of Tokyo Hospital, Hongo 7-3-1, Bunkyoku, Tokyo 113-8655, Japan; 7 Department of Pain and Palliative Medicine, The University of Tokyo Hospital, Hongo 7-3-1, Bunkyoku, Tokyo 113-8655, Japan

**Keywords:** autotaxin, genetic polymorphisms, nociceptive pain, opioid requirement

## Abstract

Autotaxin, encoded by the *ENPP2* gene, is a known key element of neuropathic pain; however, its involvement in nociceptive pain processing remains unclear. We explored the associations between postoperative pain intensity, 24-h postoperative opioid dose requirements, and 93 *ENNP2*-gene single-nucleotide polymorphisms (SNPs) in 362 healthy patients who underwent cosmetic surgery using the dominant, recessive, and genotypic models. Next, we validated the associations between relevant SNPs on the one hand and pain intensity and daily opioid dosages on the other in 89 patients with cancer-related pain. In this validation study, a Bonferroni correction for multiplicity was applied on all relevant SNPs of the *ENPP2* gene and their respective models. In the exploratory study, three models of two SNPs (rs7832704 and rs2249015) were significantly associated with postoperative opioid doses, although the postoperative pain intensity was comparable. In the validation study, the three models of the two SNPs were also significantly associated with cancer pain intensity (*p* < 0.017). Patients with a minor allele homozygosity complained of more severe pain compared with patients with other genotypes when using comparable daily opioid doses. Our findings might suggest that autotaxin is associated with nociceptive pain processing and the regulation of opioid requirements.

## 1. Introduction

In the past two decades, pain has remained one of the most frequent reasons for patients to seek medical care and remains a leading source of human anguish and disability [1,2]. Three mechanistic terms of pain are considered: nociceptive pain, neuropathic pain, and nociplastic pain. Nociceptive pain is a type of pain that involves the detection of noxious stimuli by nociceptors, and inflammatory pain is a sub-type of nociceptive pain that results from the activation and sensitization of nociceptors by inflammatory mediators. Regarding nociceptive inflammatory pain, various underlying pathophysiological mechanisms have been suggested. Among these suggested mechanisms, lysophosphatidic acid (LPA) signaling (which involves LPA), lysophosphatidylcholine (LPC) (an LPA precursor), and autotaxin (an LPA-producing enzyme encoded by the *ENPP2* gene) [3] might be involved in nociceptive pain processing. Previous studies reported that LPA is released during tissue injury or inflammatory states [4] and that LPA levels were elevated in inflammatory exudates [5]. Following a nociceptive event, an inflammatory cycle is feed-forwardly activated, since LPA increases the expression of cyclooxygenase-2 and multiple pro-inflammatory cytokines and chemokines, which in turn promote further autotaxin secretion [6]. Altered autotaxin expression has been connected to several inflammatory conditions, such as autoimmune pancreatitis, cholangitis, breast and pulmonary fibrosis, rheumatoid arthritis, and allergic asthma and dermatitis [7]. Further, under clinical nociceptive pain conditions, increased LPA levels were observed in synovial fluid samples of patients with knee osteoarthritis [8]. Thus, the autotaxin/LPA axis has implications for nociceptive pain through the inflammatory process as well as for neuropathic pain because these pain types show some similarities in their mechanistic models [9]. However, its clinical involvement in nociceptive inflammatory pain has not yet been investigated fully.

Instead of nociceptive inflammatory pain, the contribution of the autotaxin/LPA axis has been well-established and promising for neuropathic pain, because the key findings from basic investigations have been replicated in a series of clinical studies. Among LPA receptors 1–6, genetic polymorphisms of the LPA receptor 1 and possibly 3 were associated with the onset of chemotherapy-induced neuropathic pain [10]. LPA concentrations in CSF were linearly correlated with several neuropathic pain intensity levels [11,12]. Some LPA and LPC species along with autotaxin could explain the differences in clinical profiles between patients with lumbar spinal canal stenosis and patients with other neuropathic pain conditions [13]. A recent finding from a preclinical study of lumbar spinal canal stenosis clearly suggests that autotaxin is responsible for LPA production in the CSF through the autotaxin/LPA axis. Therefore, autotaxin is a potential candidate for the key regulator of neuropathic pain development and maintenance [14].

Accordingly, we focused on autotaxin in the present study and investigated its involvement in nociceptive inflammatory pain processing. Genetic polymorphisms of the *ENPP2* gene, which encodes autotaxin, are reportedly involved in the disease activity of systemic lupus erythematosus, an autoimmune disease [15]. We investigated the associations between genetic polymorphisms of the *ENPP2* gene and two types of nociceptive pain: postoperative pain and cancer pain.

## 2. Results

### 2.1. Experiment 1

A total of 93 SNPs of the *ENPP2* gene and three models in the exploratory cohort were associated with postoperative opioid dose requirements and postoperative pain intensity (visual analogue scale [VAS]: median 27.1 [inter-quartile range: 10.0–41.3]). The following two SNPs were significantly associated with opioid dose requirements. One SNP (rs7832704) demonstrated significance in the recessive (minor allele homozygosity [minor], *n* = 42; opioid dose = 3.541 [1.864–4.641] mcg/kg; *p* = 0.018) and genotypic (major allele homozygosity [major], *n* = 164; opioid dose = 2.312 [0.934–4.317] mcg/kg; heterozygosity [hetero], *n* = 154; opioid dose = 2.149 [1.081–4.067] mcg/kg; minor, *n* = 42; opioid dose = 3.541 [1.864–4.641] mcg/kg; *p* = 0.049) models. The other SNP (rs2249015) showed a significance in the recessive model (minor, *n* = 37; opioid dose = 3.750 [2.000–4.583] mcg/kg; *p* = 0.018). No SNPs including the two SNPs were associated with postoperative pain intensity (rs7832704: [recessive], *p* = 0.302; [genotypic], *p* = 0.582; rs2249015 [recessive], *p* = 0.719).

### 2.2. Experiment 2

The Bonferroni correction was applied for multiplicity, and we set the significance level at *p* < 0.017 (i.e., 0.05/3 for the three relevant models [two recessive models and one genotypic model] from Experiment 1) for the recessive model and *p* < 0.0057 (i.e., 0.017/3 for the three genotypes of the rs7832704 SNP identified in Experiment 2) for the genotypic model. The relevant *ENPP2* SNPs and their respective models demonstrated significant associations with the numeric rating scale (NRS) of cancer pain intensity, although the total daily opioid doses were comparable (Table 1).

## 3. Discussion

The autotaxin/LPA axis is a known key element of neuropathic pain, and the present genetic association studies revealed that autotaxin is involved in nociceptive pain processing, such as postoperative and cancer-related pain, which increases opioid requirements. To the best of our knowledge, our study is the first to report two relevant SNPs of the *ENPP2* gene, and the functional interpretation of these genetic polymorphisms has not yet been reported, although these SNPs demonstrated diametrically opposite aspects of postoperative pain and cancer-related pain. Both rs7832704 and rs2249015 are intronic SNPs of the *ENPP2* gene. Although both SNPs mapped approximately 400 bp away from the splice site consensus sequence in the nearest exons, the possibility that respective SNPs generate splice variants cannot be excluded. The respective SNPs may also be located in a regulatory sequence, which would increase the transcription rate. Therefore, further studies on how these intronic SNPs affect autotaxin protein function and expression levels are required in the future.

Autotaxin is the second member of the ectonucleotide pyrophosphatases/phosphodiesterase family, also known as *ENPP2*. Autotaxin contributes to LPA levels, primarily by converting mainly LPC into LPA, which exerts potent biological properties on six types of LPA receptors [16]. Indeed, plasma autotaxin levels have a close positive correlation with LPA levels [17]. The autotaxin/LPA axis plays a critical role in diverse physiological conditions, such as angiogenesis, neuronal development, and lymphocyte migration, by producing LPA [18]. The autotaxin/LPA axis has been associated with melanoma [19,20] and other tumor types [21]. Autotaxin exists not only in serum and cerebrospinal fluids (CSFs) in similar concentrations [22]; LPA and LPC also exist in CSFs [23], suggesting that the autotaxin/LPA axis may play an important role in the neural system. LPA in the CSF mediated inflammatory pain through LPA receptor 1 in a previous basic investigation [24].

Two mechanisms may explain the association between *ENPP2* polymorphisms and both pain severity and opioid requirements observed in the present study. First, the autotaxin/LPA axis can aggravate inflammation. Second, the autotaxin/LPA axis can attenuate opioid analgesia in the spinal cord after affecting the descending pain modulatory circuits.

The autotaxin/LPA axis was identified as the key regulator of lymphocyte homing and inflammation [25]. Nociceptive inflammatory pain, characterized by immune cell infiltration following tissue injury, could worsen the resulting increased opioid requirements in patients with the minor allele genotype of the *ENPP2* gene. Basic findings from a previous study clearly indicated that an enhancement of LPA signaling through LPA receptors promoted nociceptive inflammatory pain [24,26]. Considering our findings and those of previous studies, minor allele genotypes of the ENPP2 gene may enhance the functional properties of autotaxin, resulting in an increased LPA production.

A separate line of inquiry into cholestatic itch might also explain the present findings. A close association was observed between cholestatic itch intensity and serum autotaxin activity, although not opioid levels, even though mu-opioid receptor antagonists have been used to treat cholestatic itch [27]. An intradermal injection of LPA can induce itch perception [28], as LPA stimulates both transient receptor potential channel vanilloid 1 (TRPV1) and LPA receptors that are emerged on C-fiber nerve endings. Opioid dose modulation could affect LPA-induced itching and nociceptive signaling through TRPV1 and LPA receptors, respectively, in the spinal cord before the signals are transmitted to the brain. LPA attenuates the endogenous opioidergic and serotonergic descending pain modulatory circuits, resulting in pain sensitization, which physiologically suppresses the transmission of spinal pain signals [29,30]. When an opioid imbalance was introduced after cholestasis, an increased opioidergic tone resulted in cholestatic itch, likely through the decreased peripheral or central activation of mu-opioid receptors and the balance shifting towards favoring kappa opioid receptor activation [31,32]. When considering all of this anecdotal evidence together, the autotaxin/LPA axis may enhance pain transmission and attenuate opioid analgesia in the spinal cord after affecting the descending pain modulatory circuits. In this case, minor allele genotypes of the *ENPP2* gene could enhance the functional property of autotaxin, resulting in an increased LPA production.

### Limitations

This study had several limitations. Phenotypes of nociceptive inflammatory pain were inconsistent between Experiment 1 (postoperative pain) and Experiment 2 (cancer-related pain). Although the present study did not demonstrate direct evidence of such diametrically opposite aspects of postoperative pain and cancer-related pain, one possible explanation might be derived from the following aspect. Autotaxin is reportedly overexpressed in several tumor types and has been linked to tumor cell proliferation, motility, and metastasis formation [21], and such carcinogenetic properties of autotaxin might have affected our present results, especially in patients with cancer-related pain. Future investigations with larger sample sizes are required to elucidate the clinical contributions of the autotaxin activity on nociceptive inflammatory pain.

## 4. Materials and Methods

The study protocol was approved by the institutional review board at each participating hospital of our research consortium (e.g., Ethics Committee, Graduate School of Medicine and Faculty of Medicine, The University of Tokyo; approval number: G2804), and written informed consent was obtained from all participants.

### 4.1. Experiment 1

#### 4.1.1. Participants

In this exploratory study, we enrolled 362 healthy patients, who were also participants in our previous study [33], and new patients whose genotyping procedures were completed by the end of the 2019 fiscal year in Japan. Our inclusion and exclusion criteria are described in detail elsewhere [33,34]. Briefly, all of the participants (American Society of Anesthesiologists Physical Status I; age, 25.9 ± 7.6 years [mean ± standard deviation {SD}]; 125 men and 233 women) were scheduled to undergo cosmetic orthognathic surgery (mandibular sagittal split ramus osteotomy) for mandibular prognathism. The surgical and anesthetic protocols were described in detail in our previous study [34]. In accordance with the various types of clinical and experimental assessments described in our previous reports [34,35,36], in this exploratory study we simply adopted two assessments for consistency with the subsequent validation study. First, the spontaneous pain intensity at 24 h after the surgery was assessed using a 100-mm VAS, with 0 mm indicating no pain and 100 mm indicating the worst pain imaginable. Second, we assessed the postoperative fentanyl doses during the first 24 h, which were normalized to body weight. Using i.v. patient-controlled analgesia with fentanyl without continuous background infusion, bolus doses of fentanyl were administrated upon the patient’s request for 24 h after the surgery. Four patients lacked at least one of these assessments and were therefore excluded from the study.

#### 4.1.2. Genotyping Method and Statistical Analysis

The genotyping protocol is described in detail elsewhere [33,34,36]. Briefly, total genomic DNA was extracted from peripheral blood samples. Genotype data for SNPs of the *ENPP2* gene were obtained from the whole-genome genotyping results, which was performed using five types of BeadChips: HumanHap300, HumanHap300-Duo, Human610-Quad v1, Human1M v1.0, and Human 1M-Duo v3 (Illumina, San Diego, CA, USA), as described in a previous study [36]. The results were qualified and cleaned. Finally, we obtained 93 SNPs of the ENPP2 gene for the statistical analysis. The following genetic analyses were conducted using PLINK v.1.07 (http://zzz.bwh.harvard.edu/plink/index.shtml; accessed on 25 August 2020). Associations of individual SNPs with postoperative pain intensity and opioid dose requirements were estimated using logistic regression analyses. To the best of our knowledge, no study has reported associations between postoperative pain intensity and opioid dose requirements or SNPs of the *ENPP2* gene. Hence, determining which association tests should be used was difficult [37]. The allelic model, commonly used in observational research, was not applied, although we used (1) dominant (one or two vs. zero copies of the minor allele), (2) recessive (two vs. zero or one copy of the minor allele), and (3) genotypic (zero copies vs. one copy vs. two copies of the minor allele) models. For a pilot genetic association study such as the present study, a formal correction for multiple testing should not be applied based on recommendations [38,39,40]. In this exploratory study, the SNPs with *p*-values < 0.05 were selected as candidate SNPs for the subsequent validation study. The statistical analysis was performed using Prism 9 software (GraphPad Software, San Diego, CA, USA). Because all of the continuous variables associated with pain-related phenotypes were non-normally distributed, nonparametric analyses, including the Mann–Whitney U test, were primarily used to detect possible associations among the continuous variables that were associated with the pain-related phenotypes.

### 4.2. Experiment 2

#### 4.2.1. Participants

Ninety patients (age, 58.4 ± 13.4 years [mean ± SD]; 50 women; pain duration, 11.2 ± 18.8 months) with cancer pain participated in the validation study after providing written informed consent; these patients were also participants in our previous study [41]. The inclusion and exclusion criteria were described in detail in our previous study [41]. Briefly, all of the participants had cancer pain (irrespective of the originating organ and malignant lesions pathology) for longer than 1 week. Patients with cognitive dysfunction, brain metastasis, or with a suspected origin of pain other than the cancer itself were excluded. We evaluated the pain intensity using an 11-point NRS, and daily dosages of opioid analgesics were based on the body weight on the day of pain assessment. Opioid dosages were adjusted and determined by patients’ attending physicians, based on their pain complaints and impaired activities of daily living. One patient was excluded owing to missing clinical assessments.

#### 4.2.2. Genotyping Method and Statistical Analysis

The genotyping protocol was nearly identical to that used in Experiment 1. In Experiment 2, we performed whole-genome genotyping using an Omni1-Quad BeadChip (Illumina). To identify associations between cancer pain intensity and total opioid dose requirements, we examined the selected SNPs. Since false-positives and false-negatives are common in genetic association studies that use conservative adjustments (such as the Bonferroni correction), weighting the risk of both was important in the confirmatory analysis. The criterion for significance was set for all the relevant *ENPP2* SNPs and their respective models from Experiment 1 after a Bonferroni correction for multiplicity.

## 5. Conclusions

Our genetic polymorphism analysis study could suggest the possibility that *ENPP2* genetic polymorphisms are associated with nociceptive inflammatory pain intensity and opioid dose requirements, although the associations were inconsistent between two types of nociceptive inflammatory pain (i.e., postoperative pain vs. cancer-related pain). These findings might indicate that the autotaxin (and its related LPA axis) is associated with enhanced nociceptive signal transmission and increased opioid dose requirements; hence, autotaxin possibly becomes an important candidate for the development of novel drugs targeting nociceptive inflammatory pain. Further validation of these findings could facilitate the identification of patients with increased risks for nociceptive inflammatory pain as well as the application of genetic markers for the optimization of opioid doses in the clinical management of nociceptive inflammatory pain.

## Figures and Tables

**Table 1:** Associations of *ENPP2* single-nucleotide polymorphisms with cancer pain intensity and total daily opioid doses in confirmatory cohort.

		Major (*n* = 38)	Heterozygosity (*n* = 41)	Minor (*n* = 10)	*p*-Value	
					Recessive Model	Genotypic Model
rs7832704	Cancer pain intensity	5 [4–6]	6 [5–8]	8 [8–10]	0.0049 *	<0.0001 *
	Total daily opioid doses (mcg/kg)	0.364 [0.042–0.855]	0.573 [0.286–1.500]	0.308 [0.259–0.616]	*0.057*	*0.059*
rs2249015	Cancer pain intensity	5 [4–6]	6 [5–8]	6 [4.3–7.5]	0.0041 *	N/A
	Total daily opioid doses (mcg/kg)	0.349 [0.021–0.874]	0.573 [0.300–1.500]	0.279 [0.259–0.616]	*0.052*	N/A

Abbreviations: N/A, not applicable; median [inter-quartile range]. * Significant after correction for multiplicity.

## Data Availability

The datasets generated during and/or analyzed during the present study are not publicly available, but are available from the corresponding author on reasonable request.
